# <90% of the recommended weight-based volume were classified as underfilled. Blood culture results were compared between bottle characteristics using chi squared and Wilcoxon rank tests as appropriate. Results were presented to stakeholders to facilitate discussions on blood culture collection. When Less isn’t More: Pre-Implementation Data from a Pediatric Blood Culture Volume Quality Improvement Program

**DOI:** 10.1017/ash.2025.378

**Published:** 2025-09-24

**Authors:** Erica Prochaska, Aigue Anne Okogun, Christina Schumacher, Karen Carroll, Slade Decker, Alexandra Minor, Raphael Gadot, Sophia Xu, Aaron Milstone

**Affiliations:** 1 Johns Hopkins University; 2 Johns Hopkins School of Medicine; 3 Johns Hopkins School of Medicine

## Abstract

**Background:** Blood culture volume is crucial to accurate diagnosis of a bloodstream infection. Underfilling blood culture bottles decreases test sensitivity and has been associated with contaminants. Pediatric blood culture volume recommendations are patient weight-based and difficult to audit. Our objectives were to assess healthcare worker pediatric blood culture volume knowledge and to measure culture volumes during the pre-implementation phase of a blood culture quality improvement program. **Methods:** Data were collected May 2024-November 2024. Surveys were administered to healthcare personnel who regularly obtained blood cultures. To estimate the collected blood volume, blood culture bottles in the laboratory were weighed, and weights were subtracted from the averaged weight pre-filled bottles. Bottles that were **Results:** <90% of the recommended weight-based volume were classified as underfilled. Blood culture results were compared between bottle characteristics using chi squared and Wilcoxon rank tests as appropriate. Results were presented to stakeholders to facilitate discussions on blood culture collection. 65 surveys were completed. 59 (90.8%) of respondents reported receiving blood culture training. Of those who received training, 51 (78.5%) reported that they had received weight-based blood culture training. A convenience sample of 1,076 bottles were weighed, representing 38.8% of blood cultures collected. Of those, 816 (75.8%) were underfilled (median percentage of recommended volume -57.8% (interquartile range (IQR) -80.3%, -12.3%)). Only 574 (54.3%) cultures were appropriately inoculated into a pediatric bottle based on patient weight. 83 bottles (7.7%) grew bacteria or fungi, 61 (73.5%) were non-commensals. There was no association between underfilling and positivity (p = 0.47). The median percentage of recommended volume did not differ between positive and negative cultures (-60.6% and -57.3%, respectively; p = 0.92). The median percentage of recommended volume for culture growing non-commensals and commensals was -55.5% and -69.1%, respectively (p = 0.01). Stakeholder groups reported that barriers to appropriate volume included: uncertainty regarding blood culture protocols, technical issues obtaining blood and total blood draw limits. **Conclusions:** In a large, regional children’s center, the majority of weighed pediatric blood culture bottles were underfilled despite the majority of respondents reporting blood culture volume training. Fill volume was not associated with positivity, which may be due to the large proportion of underfilled bottles in this sample. Non-commensal blood cultures did have a higher median percentage of recommended volume as compared to commensal blood cultures, which is consistent with prior publications. Future quality improvement programs will focus on dissemination of policy and addressing systems and technical barriers.

